# Supplementary material for: Community-based health insurance dropout and its determinants among women in Sidama National Regional State, Southern Ethiopia, 2024: A multilevel analysis
Source: PLoS One. 2025 Aug 18;20(8):e0329382. doi: 10.1371/journal.pone.0329382 (PMC12360522; doi:10.1371/journal.pone.0329382)
Supplement: S2 File — (DOCX) [file pone.0329382.s002.docx]

**Variables measurement**

The term **age** denotes the chronological age of women as reported at the time of data collection.

**Family size** is defined as the total number of individuals residing in a household at the time of data collection.

**CBHI enrollment status** specifically indicates whether an individual was, at the time of data collection, a member of the community-based health insurance (CBHI) scheme. This status was recorded as a dichotomous variable, categorized as either "enrolled (yes)" or "not enrolled (no)," thereby clearly distinguishing between CBHI members and non-members.

The **CBHI dropout rate** refers to the proportion of women who stop participating in the program. This rate is essential for evaluating the program's sustainability and overall effectiveness.

**Women's autonomy** refers to the proportion of women who were able to make decisions regarding participation in CBHI and other livelihood matters, either independently or jointly with their husbands. Conversely, women who lacked this ability - whether independently or collaboratively - were classified as non-autonomous, when decision-making authority rested solely with male household members.

**Community-level women's autonomy** serves as a proxy measure for assessing women's decision-making power within the *kebel*e. Communities were categorized as autonomous or nonautonomous based on women's involvement in decision-making related to livelihood issues. A community was considered to exhibit women's autonomy if over 50% of women participated in decision-making processes - either independently or in conjunction with their husbands - at the *kebele* level. If this threshold is not met, indicating that fewer than 50% of women are engaged in decision-making, the community is classified as lacking autonomy.

**Community-level women's literacy** was determined by the proportion of women within a *kebele* who have achieved at least a primary level of education. If more than 50% of women have completed at least primary education, the community is classified as having a "high" proportion of literate women; otherwise, it is categorized as "low."

**Formal education** refers to structured educational programs that extend from primary through secondary and higher education, characterized by organized curricula implemented according to a defined calendar and timetable.

**Place of residence** was categorized into urban and rural classifications.

**Community-level poverty** was assessed by calculating the aggregated percentage of households within a *kebele* that belong to the poorest and poorer quintiles based on woman participant data. If this percentage exceeds 50%, the community is classified as having a "high" degree of poverty; otherwise, it is designated as "low."

**Recorded religion** refers to a classification system that categorizes women based on their religious affiliation, distinguishing between Protestants and other religious groups. This classification facilitated the analysis and understanding of religious demographics within the study population.

**Recoded ethnic group** denotes a classification system that categorizes women according to their ethnic affiliation, with those identifying as Sidama grouped in one category while other ethnic groups are consolidated into another category. This classification aided in analyzing ethnic demographics within the study population.

**Recoded education** refers to the categorization of women based on their educational background, specifically differentiating between those who have attended formal education and those who have not.

**Recoded marital status** categorizes women according to their marital status, including a category for married women and another for those with different marital statuses.

The term **employment** classifies women based on their employment status, encompassing categories for those currently employed and those who are not employed.

**Household head** categorizes households based on the gender of the household head as reported by women, distinguishing between male-headed and female-headed households.

The term **benefit packages** categorize essential healthcare offerings based on their relevance as reported by woman members of the CBHI. This categorization included both fully included benefit packages and those reported as partially included.

**CBHI premium contribution** refers to the classification of household contributions to the CBHI program based on reports provided by women. These contributions were classified as either fair or not fair, reflecting women's perceptions.

**Decisions** made by the *Woreda* Committee regarding the CBHI program can be classified into two categories: extremely transparent and somewhat transparent, based on women's reports that reflect their perceptions of transparency in the decision-making process.

**CBHI decision** **inclusiveness** refers to whether decisions made regarding CBHI were classified as either extremely inclusive of relevant actors or partly inclusive of relevant actors, reflecting women's perceptions regarding stakeholder involvement in CBHI decision-making processes.

The term **CBHI promotion adequacy** assesses the sufficiency of promotional efforts related to the CBHI program, categorizing promotional activities reported by women as either adequate or not adequate.

The term **CBHI acceptable strategy** categorizes perceptions regarding the CBHI strategy program as either acceptable or not acceptable, based on perceptions made by women within their respective communities.

The **wealth index** represents an aggregate measure of household assets calculated using principal component analysis (PCA). It was assessed through inquiries about various asset components, including livestock, crop production, infrastructure (e.g., radio, modern bed, mattress, phone, water pump, modern stove), latrine facilities, housing conditions (e.g., number of rooms, roof type, floor type), and total farm size. The wealth index was computed using PCA techniques designed to reduce dimension in large datasets.

Consequently, our PCA excluded any assets or variables held by less than 5% or more than 95% of individuals in the sample. Ultimately, component factors or wealth index scores were ranked into five classes: lowest, second-lowest, middle, fourth-highest, and highest. Variables that did not meet assumptions - such as a Kaiser-Meyer-Olkin (KMO) measure below 0.5, commonalities below 0.5, or complex structures with high loading correlations (>0.4 on multiple components) -were also removed from PCA analysis.

**Health service satisfaction:** Health service satisfaction is a composite measure that assesses patients' satisfaction in the past twelve months with healthcare services through their responses to 15 specific questions. Each question is rated on a five-point Likert scale, with 1 representing "strongly dissatisfied" and 5 representing "strongly satisfied." The overall satisfaction score is derived by summing the individual ratings, resulting in a total score that ranges from 15 (indicating all responses are "strongly dissatisfied") to 75 (indicating all are "strongly satisfied"). This quantitative method enables the assessment of health service satisfaction specifically among women’s groups.
