# Supplementary material for: Community-based health insurance dropout and its determinants among women in Sidama National Regional State, Southern Ethiopia, 2024: A multilevel analysis
Source: PLoS One. 2025 Aug 18;20(8):e0329382. doi: 10.1371/journal.pone.0329382 (PMC12360522; doi:10.1371/journal.pone.0329382)
Supplement: S3 File — (DOCX) [file pone.0329382.s003.docx]

Model selection

**Model comparison and** s**election criteria of the community-based health insurance dropouts among women in the central zone of Sidama region, Ethiopia, 2024 (n=835)**

The table presented model selection criteria for four distinct models used to analyze factors influencing dropout rates in community-based health insurance (CBHI). The criteria included log-likelihood (LL), Akaike Information Criterion (AIC), and Bayesian Information Criterion (BIC).

The null model served as a baseline, with a log-likelihood (LL) of -233.0547, an AIC of 470.1093, and a BIC of 479.5642. These high values indicated a poor fit, as this model did not include any predictors (Table 3).

In contrast, the individual-level variable model demonstrated a significant improvement over the null model, with an LL of -150.0546, an AIC of 326.1091, and a BIC of 387.5657. This model incorporated individual-level variables and showed better performance in terms of AIC and BIC, thereby suggesting it provided a more accurate representation of the data (Table 3).

However, the community-level variables model had an LL of -196.7455, with an AIC of 3405.4909 and a BIC of 433.8555. The exceptionally high AIC indicated that this model performed poorly despite including community-level predictors (Table 3).

Ultimately, the individual and community-level variables Model combined both individual and community-level variables, achieving the lowest AIC (300.4574) and BIC (361.9140), with an LL of -137.2287. This finding indicated that it provided the best fit for the data (Table 3).

Consequently, the individual and community-level variables model was identified as the best fit due to its significantly lower AIC (300.4574) and BIC (361.9140) values, which reflected a superior balance between goodness of fit and model complexity (Table 3).

In contrast, the community-level variables model exhibited extremely high AIC values (3405.4909), suggesting it did not effectively explain the data despite incorporating community-level factors. Moreover, the transition from the null model to both the individual variable model and the individual and community variable model highlighted the importance of including predictors in enhancing model performance.

In summary, the individual and community-level variables model was selected as the best fit for analyzing dropout rates in CBHI due to its optimal AIC and BIC values along with its log-likelihood (-137.2287). This model effectively captured the relevant factors while maintaining parsimony compared to the other models assessed.

**Table 3: Model comparison and** s**election criteria of the community-based health insurance dropouts among women in the central zone of Sidama region, Ethiopia, 2024 (n=835)**

| Types of the models | Model selection criteria and the results | | |
| --- | --- | --- | --- |
|  | LL (model) | AIC | BIC |
| Null model | -233.0547 | 470.1093 | 479.5642 |
| Individual level variables | -150.0546 | 326.1091 | 387.5657 |
| Community level variables | -196.7455 | 3405.4909 | 433.8555 |
| **Individual and community-level variables** | **-137.2287** | **300.4574** | **361.9140** |
