# Supplementary material for: Community-based health insurance dropout and its determinants among women in Sidama National Regional State, Southern Ethiopia, 2024: A multilevel analysis
Source: PLoS One. 2025 Aug 18;20(8):e0329382. doi: 10.1371/journal.pone.0329382 (PMC12360522; doi:10.1371/journal.pone.0329382)
Supplement: S5 File — (PDF) [file pone.0329382.s005.pdf]

ሀዋሳ ዩኒቨርሲቲ
ህክምናና ጤና ሳይንስ ኮሌጅ
የምርምር ስነ-ምግባር ገምጋሚ ቦርድ

HAWASSA UNIVERSITY
COLLEGE OF MEDICINE AND HEALTH SCIENCES
Institutional Review Board

Ref. No: IRB/021/16
Date: 22/11/2023

Name of Researcher(s): **Kare Chawicha (MSc), Mesay Hailu (PhD, Asso. Prof.), Keneni Gutema (PhD, Asst. Prof.)**

Topic of Proposal: ***The role of community based health insurance and its determinants on health services satisfaction and utilization among women in Sidama national regional state, Ethiopia***

Dear researcher(s),
The Institutional Review Board (IRB) at the College of Medicine and Health Sciences of Hawassa University has reviewed the aforementioned research protocol with special emphasis on the following points:

1. Are all principles considered?
   - 1.1. Respect for persons: Yes ☑ No ☐
   - 1.2. Beneficence: Yes ☑ No ☐
   - 1.3. Justice: Yes ☑ No ☐
2. Are the objectives of the study ethically achievable? Yes ☑ No ☐
3. Are the proposed research methods ethically sound? Yes ☑ No ☐

Based on the aforementioned ethical assessment, the IRB has:

- **A.** Approved the proposal for implementation ☑ Approval period from Nov.22/ 2023 to Nov.21 /2024
- B. Conditionally Approved ☐ -Element Approved: **Protocol Version No. 1**
- **C.** Not Approved ☐ -Follow up report expected in **6 months**

Obligation of the PI:

1. Should comply with the standard international and national scientific and ethical guidelines
2. All amendment and changes made in protocol and consent form needs IRB approval
3. The PI should report SAE within 3 days of the event
4. End of study, including manuscript should be reportedlto the IRB

Yours faithfully,

Embialle Mengistie (PhD, Asso. Prof.)
Institutional Review Board Chairperson.

☎: + 046 8209290 Website: Fax: + 046 2208755 ✉ 1560 CMHS, Hawassa-Ethio
