# Supplementary material for: Community-based health insurance dropout and its determinants among women in Sidama National Regional State, Southern Ethiopia, 2024: A multilevel analysis
Source: PLoS One. 2025 Aug 18;20(8):e0329382. doi: 10.1371/journal.pone.0329382 (PMC12360522; doi:10.1371/journal.pone.0329382)
Supplement: Attachment — Submitted filename: Response to Reviewers.docx [file pone.0329382.s007.docx]

**A step by step to the reviewers’ and editor’s comments**

**Requirement 1: PLOS ONE Style compliance and file naming**
“Please ensure that your manuscript meets PLOS ONE's style requirements, including those for file naming. The PLOS ONE style templates can be found at <https://journals.plos.org/plosone/s/file?id=wjVg/PLOSOne_formatting_sample_main_body.pdf> and
[https://journals.plos.org/plosone/s/file?id=ba62/PLOSOne_formatting_sample_title_authors_affiliations.pdf”](https://journals.plos.org/plosone/s/file?id=ba62/PLOSOne_formatting_sample_title_authors_affiliations.pdf%E2%80%9D)

**Authors’ response:**
Thank you for your guidance. We have reformatted the manuscript and all associated documents according to the official PLOS ONE style templates. All files, including the main text, figures, and supplementary materials, have been renamed to fully comply with the journal’s file naming requirements.

**Requirement 2: Consistency in grant information**
“We note that the grant information you provided in the ‘Funding Information’ and ‘Financial Disclosure’ sections does not match. When you resubmit, please ensure that you provide the correct grant numbers for the awards you received for your study in the ‘Funding Information’ section.”

**Authors’ response:**
Thank you for pointing out this discrepancy. We have thoroughly reviewed the grant information and ensured that the grant numbers and funding details are now identical and accurate in both the ‘Funding Information’ and ‘Financial Disclosure’ sections of the manuscript.

**Requirement 3: Amended funding statement**
“Please provide an amended statement that declares all the funding or sources of support (whether external or internal to your organization) received during this study, as detailed online in the guide for authors at <http://journals.plos.org/plosone/s/submit-now>. Please also include the statement ‘There was no additional external funding received for this study.’ in your updated Funding Statement. Please include your amended Funding Statement within your cover letter. We will change the online submission form on your behalf.”

**Authors’ response:**
Thank you for your suggestions. We have revised the funding statement to declare all sources of support as per the journal’s guidelines. The required sentence, “There was no additional external funding received for this study,” has been added. The amended statement was included in the cover letter as instructed.

**Requirement 4: Funding information placement**
“Please note that funding information should not appear in the Acknowledgments section or other areas of your manuscript. We will only publish funding information present in the Funding Statement section of the online submission. Please remove any funding-related text from the manuscript.”

**Authors’ response:**
Thank you for this clarification. We carefully reviewed the entire manuscript and removed all funding-related information from the acknowledgments and any other sections, ensuring that such details appear solely in the Funding Statement.

**Requirement 5: Supporting information captions**
“Please include captions for your Supporting Information files at the end of your manuscript, and update any in-text citations to match accordingly. Please see our Supporting Information guidelines for more information: [http://journals.plos.org/plosone/s/supporting-information.”](http://journals.plos.org/plosone/s/supporting-information.%E2%80%9D)

**Authors’ response:**
Thank you for your suggestion. We have added detailed captions for all Supporting Information files at the end of the manuscript and updated all in-text citations to ensure they correspond precisely to the supplementary materials.

**Reviewer #1**

**Comment 1: Dropout proportion and sampling**
“This study is interested in dropout from CBHI and its determinants. Out of 835 respondents interviewed, only 77 (9.22%) respondents who dropped were part of this study. Remaining 91% have not dropped out, and so may not provide adequate reasons for dropout. One of the major inclusion criteria for this study could have been to include mainly those who dropped out of CBHI.”

**Authors’ response:**
Thank you for your observation. We recognize the limitation inherent in the relatively small proportion of dropouts (9.22%) obtained in our study. However, the simple random sampling technique we employed ensured that both dropouts and non-dropouts had an equal likelihood of selection, thereby producing a sample representative of the broader population distribution.

Additionally, to better understand why households drop out, under a larger project umbrella which included several studies, we have conducted a separate study focusing on individuals who have left the CBHI scheme. This complementary approach allows for a richer exploration of their experiences and underlying motivations to dropout.

**Comment 2: Value of qualitative study**
“A study like this could have benefit more through qualitative study that will examine dropout rate and its determinants especially through those that dropped out and other stakeholders.”

**Authors’ response:**
Thank you for your valuable suggestion. As part of the larger project, we have already carried out a separate qualitative study titled “…Barriers and Enablers of CBHI in Sidama Region…”. This study has thoroughly addressed the suggestions and concerns raised by the reviewer.

**Comment 3: Descriptive results and tables**
“Result section of this study contained so much descriptive, knowing that such will be supported with Tables or figures.”

**Authors’ response:**
Thank you for your feedback. We have condensed the results section, reduced narrative descriptions and referred readers to the relevant tables and figures for detailed information.

**Comment 4: Socioeconomic demographics in table 1**
“Table 1 in the paper did not present the socioeconomic demographics adequately. For instance, from the sample size determination, the authors showed the region, the city administrations and urban-rural divides. These could have been presented in Table 1 to enable the reader to understand the study components.”

**Authors’ response:**
Thank you for this suggestion. We have revised Table 1 to include socioeconomic demographics, specifically adding variables for woreda, city administration, and urban/rural status.

**Comment 5: Reliability and validity distinction**
“In lines 180-182, reliability and validity of instruments cannot be achieved together through Cronbach's Alpha. The authors need to make a clear distinction between reliability and validity processes and their results.”

**Authors’ response:**
Thank you for your attention to methodological rigor. We have revised the relevant section to accurately distinguish between reliability (measured by Cronbach’s Alpha) and validity (assessed through other means), reporting each separately.

**Comment 6: Model specification and results focus**
“Again, the paper dwelt so much on the analysis model specifications than the actual results. For instance, Intraclass Correlation Coefficient, Model comparison and selection criteria etc are not very important for reader's comprehension of the study.”

**Authors’ response:**
Thank you for your suggestion. We have summarized the model specification details and shifted the focus of the results section toward the main findings and their implications. We moved the Intraclass Correlation Coefficient and model comparison to Supplementary files.

**Comment 7: Table 4 alignment with narrative**
“Table 4 and its descriptive are not aligned. While the descriptive contained the p-values, the Table itself does not contain p-values, which is very important in statistical inference.”

**Authors’ response:**
Thank you for your careful review. We have updated Table 4 and included p-values and ensured that the table content and accompanying narrative are fully aligned.

**Comment 8: repetitive statements**
“Some sentences were repeated over and over from the abstract to the result section. For example, cross-sectional analysis from December 2023 to January 2024.”

**Authors’ response:**
Thank you for highlighting this. We have edited the manuscript to eliminate repetitive statements, ensuring clarity and conciseness throughout.

**Reviewer #2**

**Comment 1: Grammatical and formatting review**
“Grammatical and formatting review: A thorough proofreading is necessary to correct grammatical errors and improve readability.”

**Authors’ response:**
Thank you for your recommendation. We conducted a comprehensive grammatical and formatting review, correcting all identified errors to improve the clarity and professionalism of the manuscript.

**Comment 2: Typographical and formatting issues**
“Typographical and formatting issues: There are instances of missing spaces, incorrect capitalization, and inconsistent reference formatting.”

**Authors’ response:**
Thank you for your attention to detail. We meticulously reviewed the manuscript for typographical and formatting inconsistencies, correcting all instances of missing spaces, capitalization errors, and reference formatting issues.

**Comment 3: Exclusion criteria detail**
“Line 210: What are other factors for your exclusion?”

**Authors’ response:**
Thank you for your request for clarification. We revised the methods section to explain exclusion criteria, providing additional detail as requested.

**Comment 4: Figure and table titles**
“Figures & Tables: Some figures (e.g., Figure 1) lack clear titles explaining what is being measured.”

**Authors’ response:**
Thank you for your suggestion. We revised all figure and table titles and legends to ensure they are descriptive and convey what is being measured or presented.

**Comment 5: Similarity between results and discussion**
“Similarity of the first paragraph of the results and the discussion.”

**Authors’ response:**
Thank you for pointing this out. We have edited both sections to ensure that the results and discussion are distinct, eliminating redundant content.

**Comment 6: Discussion on CBHI dropout rate challenges for women**
“Expanding the discussion on CBHI dropout rate challenges (particularly for women)”

**Authors’ response:**
Thank you for this valuable suggestion. We have expanded the discussion section to provide a more thorough analysis of the specific challenges experienced by women regarding CBHI dropout, including relevant literature and policy implications.

**Comment 7: Design effect in sampling**
“Your sampling technique was multistage. How did you consider the design effect?”

**Authors’ response:** We updated the methods section to explicitly describe how the design effect was considered in the sample size calculation as follows.

The sample size calculation for this study was conducted using OpenEpi 3 software, drawing on a previously reported proportion of 49% from the Tigray region, Ethiopia. The formula for proportions was employed:

$$n=\frac{Z^{2}\cdot p\cdot(1-p)}{E^{2}}$$

yielding an initial estimate of 384 participants at a 95% confidence level ($Z=1.96$) and 5% margin of error ($E=0.05$). To address clustering effects inherent in the multistage sampling design, a design effect (DEFF) of 2 was applied, consistent with World Health Organization recommendations for community-based surveys (Ramsey, 2014), resulting in an adjusted sample size of 768.

Following cluster allocation, the design effect was recalculated using the intraclass correlation coefficient (ICC). With an average cluster size of 54.93 and a conservative ICC value of 0.01 (within the 0.01–0.05 range typical for health surveys), the revised DEFF was computed as:

$$DEFF=1+(n_{\text{avg}}-1)\times ICC=1.54$$

Despite this, the original DEFF of 2 was retained to ensure statistical robustness, a conservative approach aligning with epidemiological practices to mitigate unmeasured clustering biases.

The minimum cluster count, derived from $768\times0.01=7.68$, was increased to 14 *kebeles* (the smaller administrative clusters in Ethiopia) to enhance geographic representativeness and account for heterogeneous population densities across urban-rural settings.

This adjustment also improved variance estimation stability in multistage sampling designs. A non-response adjustment of 10% ($n=768/0.9$) yielded a final sample size of 845, addressing potential participant attrition.

Methodologically, this approach demonstrates rigor through its transparent handling of design effects. The decision to retain DEFF=2 despite a lower calculated value reflects adherence to conservative statistical principles in public health research, while the ICC selection aligns with established guidelines for studies lacking prior cluster correlation data. The cluster expansion strategy balances logistical feasibility with scientific validity, ensuring findings remain generalizable to diverse communities in the region.

This systematic integration of theoretical frameworks and empirical adjustments provides a replicable model for similar studies in low-resource settings, prioritizing both precision and practicality.

**Additional Journal Instructions**

**Requirement 1:**

“PLOS authors have the option to publish the peer review history of their article (what does this mean?). If published, this will include your full peer review and any attached files.”

**Authors’ Response:**
We reviewed this option and indicated our preference regarding the publication of the peer review history during the submission process, per journal policy.

**References**

Ramsey, M.H., 2014. Sampling and sample preparation, Modern analytical geochemistry, Routledge, pp. 12-28.
