# Supplementary material for: Community-based health insurance dropout and its determinants among women in Sidama National Regional State, Southern Ethiopia, 2024: A multilevel analysis
Source: PLoS One. 2025 Aug 18;20(8):e0329382. doi: 10.1371/journal.pone.0329382 (PMC12360522; doi:10.1371/journal.pone.0329382)
Supplement: Attachment — Submitted filename: Response to Reviewers two.docx [file pone.0329382.s008.docx]

**A point–by–point response to the editorial team’s ’comments and journal requirements**

**Comment 1: Please upload a Response to Reviewers letter which should include a point-by-point response to each of the points made by the Editor and / or Reviewers. (This should be uploaded as a 'Response to Reviewers' file type.) Please follow this link for more information:** [**http://blogs.PLOS.org/everyone/2011/05/10/how-to-submit-your-revised-manuscript/**](http://blogs.PLOS.org/everyone/2011/05/10/how-to-submit-your-revised-manuscript/)

**Authors’ response:** Thank you for your guidance. We have prepared a detailed Response to Reviewers letter addressing each comment point by point. This document has been uploaded as a separate file under the “Response to Reviewers” category. We trust this will assist in the smooth evaluation of our revised manuscript.

**Comment 2: Please ensure that you refer to Figure 1 in your text, as, if accepted, production will need this reference to link the reader to the figure.**

**Authors’ response:** Thank you for reminding me. We have revised the manuscript to include a clear reference to Figure 1 in the Results section. This ensures proper linkage of the figure in the final published article.
